# Maternal Immunization: New Perspectives on Its Application Against Non-Infectious Related Diseases in Newborns

**DOI:** 10.3390/vaccines5030020

**Published:** 2017-08-01

**Authors:** Federica Riccardo, Aline Réal, Claudia Voena, Roberto Chiarle, Federica Cavallo, Giuseppina Barutello

**Affiliations:** 1 Department of Molecular Biotechnology and Health Sciences, Molecular Biotechnology Center, University of Torino, Torino 10126, Italy; federica.riccardo@unito.it (F.R.); aline.real@edu.unito.it (A.R.); giuseppina.barutello@unito.it (G.B.); 2 Department of Molecular Biotechnology and Health Sciences, Center for Experimental Research and Medical Studies, University of Torino, Torino 10126, Italy; claudia.voena@unito.it (C.V.); roberto.chiarle@unito.it (R.C.); 3 Department of Pathology, Children’s Hospital Boston and Harvard Medical School, Boston, MA 02115, USA; roberto.chiarle@childrens.harvard.edu

**Keywords:** maternal immunization, childhood cancer, neuroblastoma, DNA vaccination, cancer prevention

## Abstract

The continuous evolution in preventive medicine has anointed vaccination a versatile, human-health improving tool, which has led to a steady decline in deaths in the developing world. Maternal immunization represents an incisive step forward for the field of vaccination as it provides protection against various life-threatening diseases in pregnant women and their children. A number of studies to improve prevention rates and expand protection against the largest possible number of infections are still in progress. The complex unicity of the mother-infant interaction, both during and after pregnancy and which involves immune system cells and molecules, is an able partner in the success of maternal immunization, as intended thus far. Interestingly, new studies have shed light on the versatility of maternal immunization in protecting infants from non-infectious related diseases, such as allergy, asthma and congenital metabolic disorders. However, barely any attempt at applying maternal immunization to the prevention of childhood cancer has been made. The most promising study reported in this new field is a recent proof of concept on the efficacy of maternal immunization in protecting cancer-prone offspring against mammary tumor progression. New investigations into the possibility of exploiting maternal immunization to prevent the onset and/or progression of neuroblastoma, one of the most common childhood malignancies, are therefore justified. Maternal immunization is presented in a new guise in this review. Attention will be focused on its versatility and potential applications in preventing tumor progression in neuroblastoma-prone offspring.

## 1. Introduction

Vaccines have provided one of the most significant contributions to the control and eradication of infectious diseases ever since the late 18th century and the first successful vaccines against smallpox and poliomyelitis. A number of differing and innovative vaccination strategies have been developed since that time, meaning that many life-threatening infectious diseases, including meningitis, rabies, diphtheria, tetanus, pertussis, tuberculosis, hepatitis A, mumps, rubella, measles and varicella, have become preventable in the majority of the world’s population [1,2,3].

A crucial, although less resonant, step forward in preventive medicine concerns *maternal immunization*; a vaccination strategy designed for women of childbearing age which is able to significantly decrease susceptibility to microbial and viral infections and lower correlated mortality in infants and their mothers, thanks to the passage of maternal antibodies through placenta and milk.

The scientific world has more recently begun to conceive vaccination as a novel strategy with which to fight cancer formation, progression and spread. These types of vaccines can be therapeutic cancer vaccines or prophylactic cancer vaccines; the former are aimed at treating existing cancer by bolstering the host’s immune response, while the latter are intended to prevent cancer formation or relapse. Unfortunately, the presence of immunoedited tumor cells, diffuse tumor burden [4,5] and the negative setting of the immunoregulatory mechanisms [6], makes it difficult to succeed with cancer vaccines within the setting of advanced disease. However, redirecting cancer vaccines towards preventing tumor relapse or minimizing pre-malignant lesions may be the right direction to move forward [7]. The major goal achieved in the field of prophylactic anti-cancer vaccines so far is the prevention of cancers associated with chronic-infections, such as Hepatitis B Virus (HBV) related hepatocellular carcinoma [8] and Human Papilloma Virus (HPV) driven carcinomas [9]. Numerous studies, over the last 30 years [10], have tackled the ambitious aim of preventing initiation even in virus-unrelated cancer; this field is still very much alive with some vaccines now in preclinical phases or clinical trials [11,12,13]. It is now clear that the vaccine-driven prevention of tumor onset and expansion rests on the coordinated action of multiple mechanisms, such as the activation of cytotoxic T lymphocytes (CTLs) and CD4^+^ T-helper (Th) cell, the release of interferon (IFN)-γ and the induction of a potent humoral immune response against a specific tumor associated antigen (TAA) and a strong immune memory [5,13,14,15], which are some of the features required of a prophylactic cancer vaccine. The parallels between the key role of specific vaccine-elicited antibodies in controlling tumor progression and the induction of high antibody levels as the foundation of the maternal immunization strategy, opens up new possibilities for the use of maternal immunization to prevent the occurrence of neoplasms that typically occur in fetuses and neonates, such as neuroblastoma (NB). This review will focus on the versatility of the maternal immunization approach, highlighting the link between the concept of maternal immunization and neonatal cancer prevention and describing the new potential role of maternal immunization as a NB-fighting tool.

## 2. Maternal Immunization

### 2.1. The Original Concept

The reciprocal immune interactions occurring at the maternal–fetal interface have been well established over time [16,17] and, together with the fetomaternal microchimerism [17,18], underpins the mother’s tolerance to the fetus and have implications for the immune status of both parties [19].

A central issue for the intimate relationship between mother and offspring is the protection provided to the baby against various pathogens by the transfer of passive immunity, both before and after birth. However, women of childbearing potential are less exposed to infectious agents nowadays than they were in the past and, in some cases, may have suboptimal immune reactions to external solicitations. As a consequence, they produce fewer protective antibodies ready to be transferred to their offspring, which results in inadequate protection against various pathogens and diseases in the first period of life characterized by a budding immune system [20,21].

The last decade has seen maternal immunization emerge as a trump card with which to tackle this issue, as it has proven itself to be a safe and effective strategy for the prevention of both vertically transmitted infection risk during pregnancy and life-threatening infections in newborn infants. This is thanks to the active immunity elicited in the mother during pregnancy then passively conferred to the offspring. Indeed, the introduction of vaccination with the vaccinia virus during pregnancy in the late 19th century demonstrated the techniques ability to confer protection in young infants [22], paving the way for other successful attempts leading to the introduction of antenatal vaccines against pertussis [23], neonatal tetanus [24,25], influenza virus [26,27] and of the combined tetanus, diphtheria and acellular pertussis (Tdap) vaccine [28,29,30]. Despite the increasing prejudices against the pre-birth vaccination practice, a copious amount of data has proven that it is harmless and beneficial [31,32,33], so much so that the above mentioned vaccines are currently recommended for use on all expectant mothers (Table 1). Other vaccines, such as those against Hepatitis A and B viruses, Pneumococcus, Meningococcus, Smallpox, Varicella, Rubella and additional live-attenuated vaccines are instead tailored for pregnant women that are subjected to specific risk factors or are indicated only postpartum (Table 1).

List of vaccines already recommended during pregnancy or currently used in case of specific infection risk. *Sources:* Centers for Disease Prevention and Control (www.cdc.gov/vaccines); World Health Organization (www.who.int); updated to March 2017.

Moreover, new antenatal vaccines that are designed to potentially control infections driven by major neonatal pathogens, such as group B streptococcus, respiratory syncytial virus and cytomegalovirus, are currently in the developmental phase [34,35,36,37,38] and included in ongoing or completed clinical trials (GBS: [39,40]; RSV: [41], NCT02247726, NCT02624947; CMV: [36], NCT00133497). Other vaccines against Rotavirus and *Vibrio Cholerae* are under investigation for antenatal application [42,43]. More recently, the concern about the harmful results on the fetal brain caused by Zika virus exposure during pregnancy [44,45] triggered the effort to develop a new vaccine for pregnant women, stirring up the debate about the necessity and safety to include pregnant woman in maternal immunization clinical trials [45].

What is certain is that maternal immunization presently appears to be a unique approach in covering the gap between birth and the maturation of the immune system, when children can receive active immunization against several pathogens as per pediatric vaccination schedules (http//www.cdc.gov/vaccines/acip). It is also an example of how to take advantage of the immune interaction between mother and offspring.

### 2.2. Milestones, Claims and New Insights

The mainstay of the success of maternal immunization rests on the ability to stimulate protective innate, cell-mediated and humoral immunity in the mother and the consequent production of a high dose of specific antibodies, which confer protection to the offspring.

In humans and other mammals, systemic immunoglobulin (Ig)G produced during pregnancy, upon exposure to a pathogen through either disease or maternal immunization, are passively transferred to the fetus through the placenta with the greater amount passed during the third trimester of gestation in humans [46]. Moreover, mucosal IgG, IgA and IgM are secreted into breast milk and ingested by the newborn through lactation [47].

The mechanism of pre- and post-natal nondegradative transport of IgG, which is the major Ig isotype induced by vaccination, implicates the neonatal Fc receptor (FcRn). This specific receptor is widely expressed by the syncytiotrophoblast and gut in humans, as well as by the yolk sac endoderm and, more efficiently, by the proximal small intestine in animal models commonly used for maternal immunization studies, such as rodents [48,49,50]. Interestingly, the FcRn-mediated transfer of IgG may permit the passage of other Ig isotypes. This possibility was demonstrated in mice and humans in the IgE antibody class that can enter the circulation of offspring in the form of IgG anti-IgE/IgE immune complexes through transplacental passage and lactation from allergic mothers [51,52].

The transport of IgA and IgM occurs postnatally by retro-transcytosis via polymeric Ig receptor (pIgR) which is expressed in the apical portion of neonatal intestinal mucosa [53]. IgA can also undergo retrograde transport across the microfold cells (or M cells) in the gut-associated lymphoid tissue of infant intestines via an unknown receptor [54]. Moreover, a new means of retrograde transport of IgA, which is linked to the transferrin receptor [55,56,57], also known as CD71, has been identified.

Another member of the immunoglobulin family to be unexpectedly transferred from mother to offspring is the IgD antibody class. Evidence for this was demonstrated a long time ago by a number of studies showing the presence of maternally-derived specific IgD in amniotic fluid, cord blood and breast milk following maternal immunization against Rubella [58,59,60]. Despite the mechanism of IgD passive influx during and after pregnancy still being unknown, the finding concerning the materno-fetal transfer of IgD early in the phylogenetic evolutionary process [61] suggests that IgD is significantly involved in the protection of the newborn. For example, this class of antibody can supply to mucosal immunity in the case of IgA deficiency and can potentiate immune surveillance by activating proinflammatory programs against pathogens of the respiratory tract [62].

The IgE antibody isotype was also found in the amniotic fluid [63], justifying the interest in their role in awaking the immune system against parasites after birth.

Overall, the Ig component of breast milk allows mucosal defense against harmful bacteria of the luminal tract to occur by direct neutralization, inactivation of toxins and other virulent factors and the inhibition of their adherence to epithelial cells. Moreover, the antibodies present in milk can transport some antigens across the neonatal intestinal mucosa with the involvement of mucosal dendritic cells (DCs), which bind IgA- or IgG-transporting antigen via FcαRI or FcγRs, inducing both immunity against pathogens and tolerance towards commensal microbiota [47,64,65,66]. This is in line with the idea that maternal immunization, rather than neonatal vaccination, can favor immune adaptation to microbial colonization shortly after birth, while avoiding any disturbance to the intestinal host-microbe homeostasis [47,67,68,69,70].

Conversely, some evidence has highlighted the inhibitory role that maternal antibodies can play in the generation of an effective humoral response in offspring following active immunization. The inhibition of seroconversion after vaccination has been shown in several cases of offspring from vaccinated mothers, such as those against measles [71,72], HBV [73], pneumococcus [74], tetanus and pertussis [74,75,76,77]. However, the inhibition of B cell responses by maternal antibodies shall cease with the decline of maternal antibodies in offspring sera [78,79], suggesting that monitoring maternal antibody concentration would allow the correct timing for vaccination in infants to be scheduled. This problem might be overcome by a number of strategies, such as an increase in the vaccine antigen dose [80,81], and other approaches which have been comprehensively reviewed [82]. Moreover, it has been recently demonstrated that heterologous maternal-infant immunization can abolish the negative effects of maternal antibodies on offspring immune responses [83,84]. This is in line with observations about the protective role of plasmid DNA-based vaccines encoding for viral antigens in infant animals born from mothers immunized with a different vaccine formulation [85,86].

### 2.3. Maternal Immunization can Confer Active Immunity to Offspring via Breastfeeding

Evidence proving that immunization during or after lactation results in better humoral and cellular immune responses in children who have benefited from breastfeeding [87,88,89], point to the role that maternal milk has to play in active immune simulation in infants. One of the mechanisms proposed to explain this issue is the passage of anti-idiotype maternal antibodies against pathogen antigens that can specifically prime an infant’s immune system [87,90]. Moreover, the presence of cytokines such as IFN-γ in maternal milk can contribute to Th2-like bias shaping the neonate immune system [91,92]. The most recent findings [93] have demonstrated the protective role that maternally derived cytokines, such as tumor necrosis factor (TNF)-α, IFN-γ, interleukin (IL)-6, IL-8 and IL-12/IL-23p40, play in a pertussis model. The authors showed that the passive transfer of cytokines during the suckling period in piglets fed by mothers immunized with heat-inactivated *Bordetella pertussis*, substantially contributes to protecting offspring, possibly by regulating their immune repertoire [93].

In addition, the presence of live activated leukocytes, including neutrophils, macrophages and lymphocytes [94], has been categorically demonstrated in milk. Interestingly, maternal lymphocytes do secrete specific factors that are able to support the humoral immune response in newborns in both T-cell dependent and independent pathways [95]. After maternal transfer, lymphocytes are taken up and found in the intestinal mucosa and Peyer’s patches in neonates upon breastfeeding [96,97] where they can induce active immunization as demonstrated in lambs fed by sheep mothers immunized against tetanus [98]. Moreover, Bandrick et al. [99] have demonstrated for the first time that colostrum-derived lymphocytes, passively transferred from vaccinated mothers to their offspring, are able to proliferate and participate in a functional response to a *Mycoplasma hyopneumoniae* antigen. Indeed, they showed an antigen-specific in vivo delayed-type hypersensitivity response in breastfed offspring and lymphocyte proliferation in vitro [99].

Furthermore, the active immunization of newborns can occur via the passage of antigens associated to specific IgG, as demonstrated in experimental models in various studies and described in the next paragraph. Immune complexes (ICs) formed by antigen and specific IgG can undergo FcRn-mediated uptake [100] at the intestinal mucosal surface. They will be shipped into vesicles, where processing of the complexed antigen releases peptides that are loaded onto MHC class I (MHC-I) and class II (MHC-II) molecules of antigen presenting cells, thus stimulating the activation of cognate CD8^+^ and CD4^+^ T cells.

Overall, the evidence described so far make maternal milk and breastfeeding irreplaceable immunity sources which confer passive immunity and actively stimulate the infant immune system.

## 3. Maternal Immunization can Prevent Non-Infectious Diseases in Offspring

The effectiveness of maternal immunization against infectious harmful agents has been largely proven and this research field is still actively productive. However, new approaches have been explored which have maternal immunization against non-infectious antigens as their main focus, further expanding upon the versatility of this vaccination strategy.

A curious and elegant approach was devised by Yamashita et al. [101] during the study of maternal immunization against an antigen prevalent in atherosclerotic lesions (oxidized LDL; oxLDL) which was performed in rabbits and mice with the aim of preventing in utero atherogenic programming driven by maternal hypercolesterolemia. They displayed a significant reduction in atherogenesis in adulthood in animal offspring born from anti-oxLDL immunized mothers and explained this effect by the passage of specific ICs, containing anti-OxLDL IgM and LDL, to the fetus. These ICs were able to remove LDL particles from fetal blood circulation and trigger specific IgM and IgG responses upon challenge with oxLDL in adult animals born from immunized mothers [101]. In parallel with this study, the same group demonstrated the inhibition of the in utero programming of diabetic conditions in mice following maternal immunization with OxLDL [102]. This approach has achieved to eliminate the risk of insulin resistance and type-2 diabetes in both mother and offspring, consistent with a regulatory effect on the expression of genes relevant to diabetes and oxidative stress [102].

A more consistent number of investigations in rodent models has focused on preventive vaccination against allergens that make use of maternal immunization. One of these studies has demonstrated how the vaccination of female mice with OVA and Al(OH)_3_, used as an adjuvant, during pregnancy and soon after delivery, can induce OVA-specific IgE suppression in offspring which received the same allergen as young adults compared to those from non-immunized mothers [103]. In contrast to IgE, the level of allergen-specific IgG2a was found to be unaffected in the offspring of immunized mothers, as compared to controls, after OVA-immunization in the presence of an adjuvant [103]. Other studies have observed IgE inhibition in the offspring of allergen-immunized mothers but an enhanced IgG response [104,105]. This evidence can be explained by the hypothesis of the induction of allergen-specific Th1-like immunity in offspring. These effects may be translated to a reduced incidence of allergy thanks to the crucial role that IgE plays in mediating the mechanism of the type-I hypersensitivity response. Indeed, independent studies have demonstrated an inhibitory effect on the development of allergic reactions and asthma in neonates exposed to OVA after the immunization of their mothers with the same allergen [104,106,107]. One proposed mechanism for the effectiveness of maternal immunization in preventing neonatal allergy was the upregulation of the expression of the inhibitory receptor FcγRIIB on offspring B cells, leading to the inhibition of B cell proliferation, avoiding skewed Th2 responses and therefore the development of allergic disorders [108].

As in the case of atherosclerosis, mentioned earlier, the prevention of allergy in mouse models required the formation and transfer of specific ICs by breastfeeding. This concept has been well described by Mosconi et al. [105], who showed that maternal exposure and sensitization to OVA during lactation induced oral tolerance in breastfed pups, preventing allergic asthma development thanks to the uptake of specific IgG1-OVA ICs. The protection conferred against allergic airway inflammation in the progeny of antigen-exposed sensitized mothers was dependent upon the expansion of OVA-specific T regulatory (Treg) cells and persisted up to 14 weeks after birth, at which point maternal IgG had disappeared from circulation, demonstrating the induction of an active tolerance to the antigen [105]. These data are in accordance with other studies that have demonstrated the induction of tolerance towards allergens upon maternal immunization which is caused by the presence of high levels of regulatory cytokines in the amniotic fluid [109]. Moreover, the transfer of specific IgG1 antibodies from mother to offspring is able to inhibit anaphylactic IgE and IgG1 antibody responses in immunized offspring [107]. It has been also demonstrated that preconceptional immunization determines the profile of DC and Treg generation in the progeny [109,110,111]. The generation of regulatory or memory T cells may explain the long-lasting effect of the tolerance status of offspring.

This abundance of evidence has now made it clear that the liability of the immunological shaping of the progeny is delegated to the mother before and in the period soon after birth. Moreover, the immunological link established between mother and offspring during pregnancy and lactation has been proven to be an invaluable source of protection for the newborn, even against non-infectious diseases. On those grounds, we believe that this complex and unique relationship can be exploited for the immune prevention of childhood cancer.

Sandler and colleagues [112] performed the first attempt at this goal via the passive immunization of female rats, before mating, with polyclonal IgG directed against the soluble 53 kDa (s53) TAA. They demonstrated that maternal passive immunization conferred protection against dimethylbenzantracene-induced tumorigenesis in offspring [112]. The reduced risk of tumor formation noted in the adult rats born from vaccinated mothers was due to the direct stimulation of their immune system, as proven in a subsequent study [113] by the expansion of the progeny’s splenic follicles and germinal centers following s53 maternal immunization.

Although lacking somewhat from the conceptual point of view, because of the passive immunization technique used and the poor translational value of the dimethylbenzantracene model, these studies represent a first step towards the application of maternal immunization to prevent cancer in offspring.

## 4. Maternal Immunization Against Tumor Associated Antigens: The Potential of DNA Vaccination

Despite the wealth of positive clinical evidence demonstrating the safety and efficacy of maternal immunization for the prevention of infectious diseases, including infection-related tumors in newborn children, its use as active immunization against TAA is not an overly explored field. Indeed, very little preclinical effort to prevent congenital tumors by maternal vaccination has been made so far.

In the last century, the characterization of several TAA in non-infection-related cancers [114], together with evidence that immune responses against these antigens could be spontaneously mounted by a patient’s immune system [115,116,117,118], has provided the rationale for the development of specific anti-TAA vaccines for cancer management. However, despite the encouraging results in preclinical studies [119,120,121,122,123,124], the overall clinical benefit has been so far limited [125].

In terms of clinical setting, one problem is that anti-cancer vaccination studies are commonly carried out when high tumor load is already present, bringing with it all the immunosuppressive phenomena that can impair and/or elude the immune response. Studies are therefore moving into earlier disease stages, since true primary cancer prevention is still a futuristic goal [15,125] and the removal or avoidance of cancer risk factors by vaccinating healthy individuals for the prevention of non-infectious related cancers is still far from being reality. However, the repositioning of preventive anti-cancer vaccination into the field of maternal immunization could renew the original concept and pave the way for a pioneering approach to the management of congenital tumors.

Among the TAA-targeted vaccination strategies, DNA mediated immunization is the youngest but most-rapidly developing technology in the immunology field. Since the characterization of the first tumor antigen, named melanoma antigen (MAGE)-1, in 1991 [126], the identification of a growing number of TAA has prompted the extensive evaluation of targeted DNA vaccines for the treatment of cancers in both preclinical and clinical studies [127,128,129]. DNA vaccination brings with it several advantages over other immunization techniques, among them, the ability to induce a complete and robust humoral and cellular immune response [5]. Moreover, more logistic advantages of DNA vaccines include the relative ease and low cost of production and transportation as well as the possibility of being applied to a wide population, independently from the immune histocompatibility. However, only a few steps forward have been taken along the path of translating DNA vaccination to the field of maternal immunization.

Some evidence has suggested that neonates, born from DNA-immunized mothers, can elicit effective immune responses against viruses even in the presence of potentially inhibitory maternally-derived Ab. The explanation probably lies in the ability of DNA plasmids to directly transfect cells in vivo and induce antigen expression after the physiological decline of maternal antibody levels. Consequently, the viral antigen is not readily available to maternal IgG binding, which may induce a cross-link between FcγRIIB (engaged by antigen specific IgG) and the B Cell Receptor (which recognize the same antigen) on B cells, thus inhibiting their activation [82].

Independent studies have demonstrated the efficacy of DNA immunization, as applied in both mothers and offspring, in priming the offspring immune system against the influenza virus [130,131,132], herpes virus [133,134], measles virus [135], rabies and pseudorabies virus [86,136]. The most recent finding in this field has demonstrated that preconceptional DNA immunization against an HIV-1 antigen (the LAMP/gag DNA chimeric vaccine) was effective for the transferring of high levels of maternal Ig, via the transplacental and breastfeeding routes, to offspring. This event leads to the temporary inhibition of the offspring immune response when vaccinated with the same construct, but surprisingly it does not affect later T- and B-cell responses, demonstrating the granting of immunological memory to the pups. Therefore, maternal DNA vaccination was found to be a good strategy for effective maternal and newborn vaccination, even if further investigations are needed to set up an effective and safe immunization protocol. All this knowledge could be successfully translated into the field of maternal immunization against TAA for the prevention of neonatal tumors.

### 4.1. Maternal Immunization can Confer Anti-Tumor Immunity Against Her2-neu

The stepping-stone in the development of a successful anti-tumor DNA vaccine is the identification of the best target antigen. Several studies have attempted to define the ideal features of a TAA to be used for DNA vaccination. Our group coined the term “oncoantigens” [5] for TAAs that drive the progression of a neoplastic lesion from one stage to the next and can be expressed on the membrane or in the cytoplasm of a tumor own cell, or be secreted by the non-neoplastic cells surrounding the tumor that form the tumor microenvironment [6,137]. Oncoantigens can be classified into three classes, according to their localization: (i) class I, antigens expressed on the cell surface; (ii) class II, antigens of the tumor microenvironment; (iii) class III, antigens confined in the intracellular compartment [138]. Some selected characteristics of oncoantigens make them useful targets for immunotherapy. First of all, the stable expression of oncoantigen throughout the various tumor development stages makes them unsusceptible to immunoediting, with the impairment of tumor progression if they come lost. Moreover, the common and high expression levels in transformed cells and/or within the tumor microenvironment, coupled with poor expression in normal cells, is a crucial feature. Finally, the susceptibility of an oncoantigen towards both T cell-mediated and antibody responses is an appealing feature [138].

Of the well-known and characterized TAAs identified so far, Her2-neu (neu) is considered to be an “ideal” oncoantigen for cancer immunotherapeutic studies. The efficacy of DNA vaccination against neu was studied exploiting a preclinical mouse model of neu-positive breast cancer called BALB-neuT [139,140,141,142]. They are BALB/c mice heterozygous for the transforming form of the rat neu transgene under the transcriptional control of the mouse mammary tumor virus promoter; then predestined to develop mammary tumors in all their mammary glands [139]. Our group demonstrated that anti-neu DNA vaccination effectively inhibits carcinogenesis in BALB-neuT female mice, with better results when followed by electroporation [143,144,145,146]. The success of this DNA electrovaccination technique rested on the induction of a strong anti-neu antibody response [143,147].

Since the success of maternal immunization relies on the same principles, the evaluation of the efficacy of active DNA maternal immunization against neu has recently been performed in order to hamper spontaneous mammary tumor progression in BALB-neuT offspring [148]. Importantly, maternal immunization against neu has proved to be well tolerated, without any side effects reported to both mother and offspring during and after pregnancy [148]. Indeed, despite the expression of neu in the embryonic tissues [149], the fertility ratio, the number of the litter and newborn size were not affected in the case of maternal immunization against neu [148]. Possible explanations underlying this phenomenon are the larger amount of antibodies passed through lactation in rodents compared to the transplacentar passage [48] and the evidence that the specific anti-rat neu antibodies induced by DNA vaccination are not cross-reactive to the murine orthologue molecule [150].

The significant extension of tumor-free and overall survival observed in BALB-neuT offspring born from and fed by mothers electrovaccinated against neu, as compared to those from control mothers, was related to the passive transfer of maternally-derived anti-neu IgG to newborns [148]. More specifically, the most abundant IgG subclass found in the milk and sera of neu-vaccinated mothers and in the sera of their pups have been demonstrated to be IgG2a [148]. Clearly, maternal immunization against neu is able to activate Th cells producing IFN-γ, the primary switch factor for IgG2a, according with previous findings [151]. Since IgG2a activate the complement and interact in an efficient manner with the Fcγ receptors on various effector cells [152], it is conceivable that upon maternal immunization against neu, important immune reactions, such as antibody-dependent cell-mediated cytotoxicity, may take place borne by the offspring. This hypothesis was confirmed by the failure of maternal immunization against neu in protecting BALB-neuT female mice from tumor progression when they were born to and fed by FcγRI/III-knock out BALB/c mothers [148].

However, the anti-tumor protective effect recognized in BALB-neuT offspring after maternal immunization was also due to an active immune response triggered in newborns against the neu oncoantigen. Indeed, even if tolerant of the neu immunodominant peptide because of its expression in the thymic stroma as a self-peptide [153], BALB-neuT offspring born from and fed by vaccinated mothers were able to develop an in vivo cytotoxic response against the same peptide (p63–71). The observation of the expansion of specific CD8^+^ T cells bearing the specific T cell receptor rearrangement against the p63–71 BALB-neuT offspring born from and fed by vaccinated mothers left out the possibility of the passive transfer of this reactive pool of T cells [148].

Moreover, neu-specific IgM^+^ memory B cells have been found in the spleens of offspring from vaccinated mothers as compared to control offspring. Indeed, the pups displayed anti-neu IgM in their sera despite the little amount of this antibody class revealed in the milk of their vaccinated mothers.

**Figure 1:**
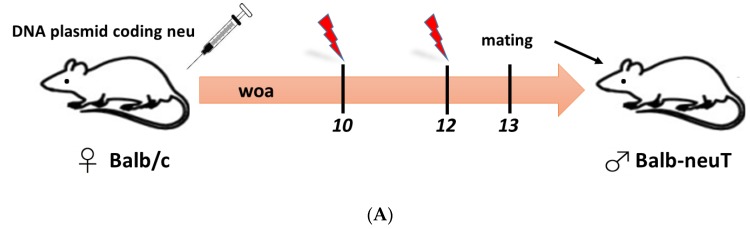

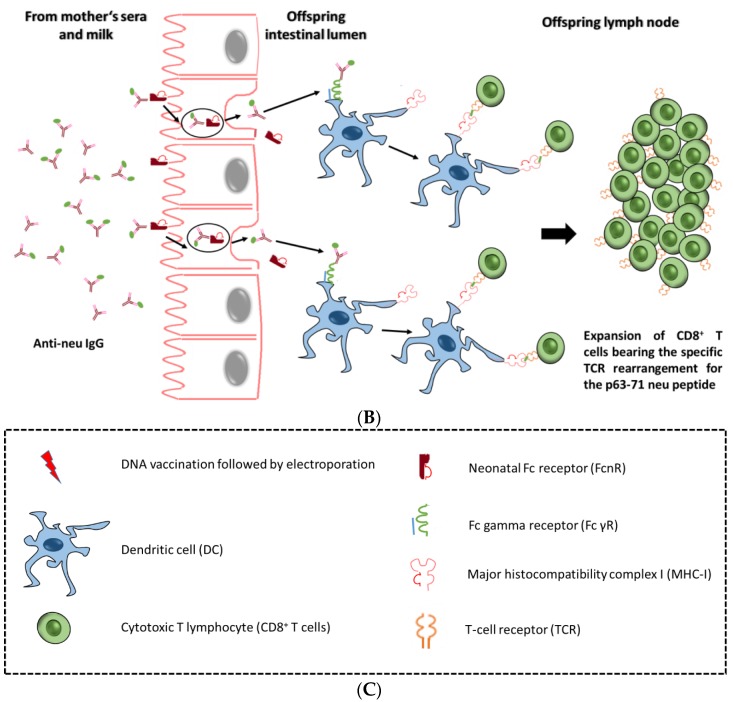
Schematic representation of the mechanism underlying maternal immunization-induced immune protection in offspring. (**A**) Maternal immunization schedule providing a prime-boost DNA electrovaccination strategy in BALB/c female mice, which then mated with a transgenic BALB-neuT male. (**B**) Maternal DNA immunization leads to high levels of anti-neu IgG antibodies in mother’s sera being passed to offspring mainly through colostrum and milk. Maternally-derived IgG alone, or complexed with the EC portion of neu, bind the FcRn on the surface of the pup’s enterocytes. The antibody-receptor complex can be internalized and then released into the intestinal lumen. The IgG-neu ICs interact with DC through Fcγ receptors. The DC can then internalize the ICs and load neu peptides onto the MHC-I or MHC-II. The binding of CD8^+^ T cells that express specific TCR against p63-71 with the MHC-I-neu peptide complex on DCs leads to the expansion of this specific CD8^+^ T cell clone into the lymph nodes of offspring born from and fed by anti-neu vaccinated mothers, activating an effective cytotoxic T cell response. (**C**) List of pictures and related abbreviations.

The activation of immune responses in offspring upon maternal immunization against neu could be explained by the principle of DNA vaccination. After DNA electrovaccination against neu, the transfected muscle cells of immunized mothers produce, by themselves, the neu protein that then could be shed [154], delivering the extracellular portion of neu protein (EC) to the blood stream. According to the FcRn-mediated mechanism described above, the anti-neu vaccine-induced IgG and the EC portion were transferred, in the form of specific ICs, to the offspring in the suckling period [146], stimulating the expansion of a specific T cell response following the cross-presentation of EC peptides onto the MHC-I and MHC-II expressed by DCs (Figure 1). Results reported in this study demonstrated that maternal immunization has the potential to hamper mammary cancer in genetically predestined offspring, in a prototype preclinical model. These findings have stimulated enthusiasm for the use of maternal immunization against oncoantigens towards the prevention and/or treatment of lethal neonatal cancer diseases.

## 5. The Rationale for the Application of Maternal Immunization Against Childhood Cancer

Neonatal cancer comprises a heterogeneous group of neoplasms. Almost all types of pediatric cancer are rare but can occur early in fetuses and neonates, ruling out the possibility of major lifestyle-related or environmental risk factors that may influence the onset of cancer as can happen in many cancers that arise in adulthood. Nevertheless, maternal exposure to risk factors may be an indirect cause of neonatal cancer [155,156].

About 10% of cancers that arise in childhood are related to a cancer predisposition syndrome [157,158,159] that may be associated with developmental defects [160]. This proportion might readily increase with advances in the understanding of the genetics of cancer. This being said, it would not be surprising if childhood cancers were found to have a prenatal origin. Indeed, they are often the result of DNA mutations in cells that sometimes even take place before birth [161]. A unique characteristic of certain childhood cancers is the development of hyperplasia in embryonic cells that has a natural disposition to spontaneously regress through cell death [162,163]. In some instances, some embryonic cells can resist the cell death signals thanks to trophic factor withdrawal, which normally occurs for deleting cells that are in excess after the completion of organogenesis [161]. These cells can later acquire additional alterations leading to malignant transformation and cancer progression.

According to the American Cancer Society, the most common cancers diagnosed in children are leukemia, lymphoma (including both Hodgkin and non-Hodgkin), Wilms’ tumor, rhabdomyosarcoma, retinoblastoma, bone cancer, brain and other central nervous system tumors and NB (http://www.cancer.org/cancer/cancerinchildren/). Of these, NB, Wilms’ tumor, retinoblastoma and B-cell lineage acute lymphocytic leukemia have been identified as arising prenatally [161].

The identification and diagnosis of such types of cancer might thus occur before birth via direct detection on ultrasound [164,165], magnetic resonance imaging (MRI) [166,167] or indirectly in view of fetal complications. Moreover, it can be argued that prenatal diagnosis of cancer will soon come from next generation sequencing technology, as has already happened for aneuploidy and other abnormalities [168,169,170,171,172,173]. Either way, genetic testing to characterize a heritable germline mutation of cancer susceptibility genes may be required [174,175], especially when there is a risk of perinatal malignancies strongly associated with a cancer predisposition syndrome.

The therapeutic decisions made when managing childhood cancer must consider the vulnerability of neonates, as well as the type and stage of the cancer, and should avoid aggressive therapies. The standard therapeutic options for pediatric cancer have included surgery, radiation-based strategies and chemotherapy for a long time, sometimes used in combination to achieve better results. Although these standard approaches lead high response rates in some cases [176,177,178], the progress in their use has reached a plateau over the last decade. Moreover, chemo-resistance and toxicities associated with increasing doses of some conventional drugs block the way to further therapy improvements and, in most cases of advanced disease, do not lead to a favorable outcome [160].

Immunotherapy approaches that have recently shown increasing promise in the field of adulthood cancer therapy seem to be viable options for improving the treatment of childhood cancer by means of chimeric monoclonal antibodies [179,180], chimeric antigen receptor T cells [181,182,183,184], and cancer vaccines [185,186,187]. Similar to the results in adults, the first two approaches are not free of side effects [182,188,189,190], while pediatric cancer tumor vaccines are safe to administer but tumor regression has so far been rarely observed [186,187,191]. These kinds of treatments could become an interesting approach in the setting of minimal residual disease burden and diminish recurrence in high-risk cases. However, the aggressive nature of many infant cancers could make the slowing of the tumor growth rate challenging even without contributing to meaningful clinical benefits. Therefore, it becomes clear that a readily workable prevention strategy for childhood malignancies is not available at the time. For this reason, it would be of great interest to develop a strategy of prevention within the antenatal setting.

### Towards the Application of Maternal Immunization against Neuroblastoma

Of the known childhood embryonal malignancies, NB is one of the most commonly diagnosed in the first year of life [192]. It is an extracranial solid tumor arising from the sympathetic nervous system with a wide range of clinical phenotypes [193]. NB accounts for sporadical or familial cases, being responsible for 15% of cancer-related deaths in children. A germline mutation in the anaplastic lymphoma receptor tyrosine kinase (ALK) gene is known to be the most common cause of hereditary NB [194]. On the other hand, familial NB is rarely associated with congenital central hypoventilation syndrome, which is caused by a germline mutation of the PHOX2B gene [195]. Approximately 10% of sporadic NB carry somatic ALK-activating mutations and an additional 4% have a high frequency of ALK gene amplification. The mutations result in constitutive phosphorylation of ALK receptor tyrosine kinase, leading to dysregulation of cell signaling and uncontrolled proliferation of the ALK-mutant neuroblasts [196]. Of several somatic activating ALK mutations identified in NB, one of the most common is a cytosine-to-adenine change in the exon 23, resulting in a phenylalanine-to-leucine substitution at codon 1174 (F1174L) within the kinase domain [194,196]. This mutation is preferentially associated with another recurrent alteration described in patients with NB and typically linked to high-risk NB [197]: the amplification of MYCN, an oncogene belonging to the MYC family of transcription factors which codes for a pleiotropic nuclear phosphoprotein [198].

Recently, Berry et al. [199] have demonstrated that ALK^F1174L^ mutation potentiates the oncogenic activity of MYCN in NB by using a transgenic mouse model that overexpresses the ALK^F1174L^ and MYCN genes in the neural crest under the control of the rat tyrosine hydroxilase promoter. These mice (ALK^F1174L^/MYCN mice), hemizygous for both human ALK^F1174L^ and MYCN on the C57/BL6J background, exhibit high tumor penetrance and rapid lethality due to the development of large and locally invasive thoracic and abdominal masses that arise in the paraspinal ganglia or adrenals [199].

According to the evidence that mutation and rearrangement imputable to ALK are associated with other malignancies, including non-small cell lung cancer (NSCLC) [200], anaplastic large cell lymphoma (ALCL) [201] and other types of neonatal cancer such as rhabdomyosarcoma [202,203,204,205], a recently reported type of congenital lung lesion (fetal lung interstitial tumor) [206] and inflammatory myofibroblastic tumors [207], the inhibition of the aberrant ALK kinase function could be a viable therapeutic option for different pathologies, besides pediatric NB. Crizotinib, a dual-specific inhibitor of ALK and c-met [208] has been recently approved by the Food and Drug Administration (FDA) for the treatment of NSCLC, and could be used against pediatric tumors. Nevertheless, the main challenge in its use is the development of therapeutic resistance. As regards the treatment of NB, acquired Crizotinib resistance has been demonstrated in specific ALK mutations, including the F1174L mutation [208,209].

Interestingly, recent preclinical studies indicate that ALK fulfills the major requirements for an ideal OA for lymphoma vaccination [210] and that a vaccine against ALK can induce a strong specific immune response able to impair the growth of ALK-rearranged lung tumors [211].

These premises have driven our idea to apply the antenatal DNA vaccination approach against ALK on the ALK*^F1174L^*/MYCN engineered transgenic mouse model on BALB/c background, generated by Prof. Roberto Chiarle in our Department. The aims of this project are to induce a strong immune response against ALK in DNA electrovaccinated mothers in order to transfer anti-tumor immunity to their cancer-prone offspring. Provisional data that we have obtained points to a trend of increasing survival and slower tumor growth kinetics in offspring born from anti-ALK vaccinated mothers, as compared to those from control mothers, accompanied by a specific antibody response against ALK borne by mothers and offspring. Nevertheless, further experiments are required before any significant conclusions can be drawn.

Noteworthy, the humoral immune response triggered by anti-ALK electrovaccination in mothers does not interfere with the pregnancy and allows the delivery of healthy pups, as in the case of maternal immunization against neu. The safety of ALK targeting was anticipated by the evidence that no altered phenotype results from ALK knockout mice [212]. Moreover, despite the expression of ALK in fetal central and peripheral nervous systems, the level of both ALK messenger RNA and ALK protein decreases to very low levels in newborns [201]. These evidences clear away the concern about the safety of maternal immunization against properly selected oncoantigens in mouse models, laying the foundations for the future translation of this approach to humans.

## 6. Conclusions

Maternal immunization has so far proven itself to be a potent tool in preventing infectious diseases in both the mother and offspring. Interestingly, a new scenario—that of exploiting maternal immunization approaches against pathologies that are not associated with infectious episodes, such as pediatric cancer—has been opened up. The proof of concept outlining the anti-tumor immunity conferred against mammary cancer progression in BALB-neuT mice has shed light on the opportunity provided by this new objective.

NB is one of the most common childhood malignancies. Conventional treatments provided to NB-affected patients currently include induction chemotherapy, surgery, high-dose chemotherapy followed by autologous stem cell reinfusion and radiotherapy. However, acute and persistent toxicities go hand-in-hand with current treatments. For these reasons, the prevention of the onset and/or progression of neonatal NB by means of maternal immunization appears to be an attractive perspective that could be translated to pediatric oncology.

## Figures and Tables

**Table 1:** Summary of recommended vaccines for pregnant and postpartum women.

Target Population	Vaccine	Type/Form	Recommendation
*All pregnant women*	Influenza	Inactivated	1 dose administered during flu at any gestational ages
	Tetanus, Diphtheria and acellular Pertussis (Tdap)	Toxoid/inactivated bacteria	1 dose ideally between 27 and 36 weeks of gestation
*Pregnant women with specific risk factors*	Hepatitis A	Inactivated whole-cell viral	2 doses; allowed in some circumstances
	Hepatitis B	Inactivated viral recombinant subunit	3 doses; allowed in some circumstances
	Pneumococcal	Inactivated bacteria polysaccharide	1 dose if there is risk factor
	Meningococcal	Inactivated bacteria polysaccharide	1 dose if there is risk factor
		Conjugate	
	Yellow fever	Live-attenuated viral	1 doses during epidemics and in case of travel to endemic regions. (Should be avoided during breastfeeding)
	Japanese Encephalitis	Live-attenuated viral	1 doses during epidemics and in case of travel to endemic regions
	Typhoid	Live-attenuated bacterial recombinant	Insufficient data for recommendation
	Anthrax	Inactivated subunit	Post-exposure prophylaxis; pre-exposure prophylaxis is not recommended
	Rabies	Inactivated whole-cell viral	Post-exposure prophylaxis; consider pre-exposure prophylaxis if risk of exposure is very high
	Tetanus and Diphteria (Td)	Inactivated bacterial toxoids	Allowed in some circumstances (Tdap preferred)
	Smallpox	Live-attenuated viral	Post-exposure prophylaxis; pre-exposure prophylaxis is not recommended
*Postpartum women (contraindicated in pregnancy)*	MMR (Measles, Mumps, Rubella)	Live-attenuated viral	1 dose immediately postpartum if susceptible to rubella
	Varicella	Live-attenuated viral	1 dose immediately postpartum if susceptible
